# Supplementary material for: Causal Relationship between Aging and Anorexia Nervosa: A White-Matter-Microstructure-Mediated Mendelian Randomization Analysis
Source: Biomedicines. 2024 Aug 16;12(8):1874. doi: 10.3390/biomedicines12081874 (PMC11351342; doi:10.3390/biomedicines12081874)
Supplement: Supplementary file 1 [file biomedicines-12-01874-s001.zip › Supplementary Figure/Figure S4/Singe SNP plot of MR analysis for TL to ubm-b-1938.pdf]

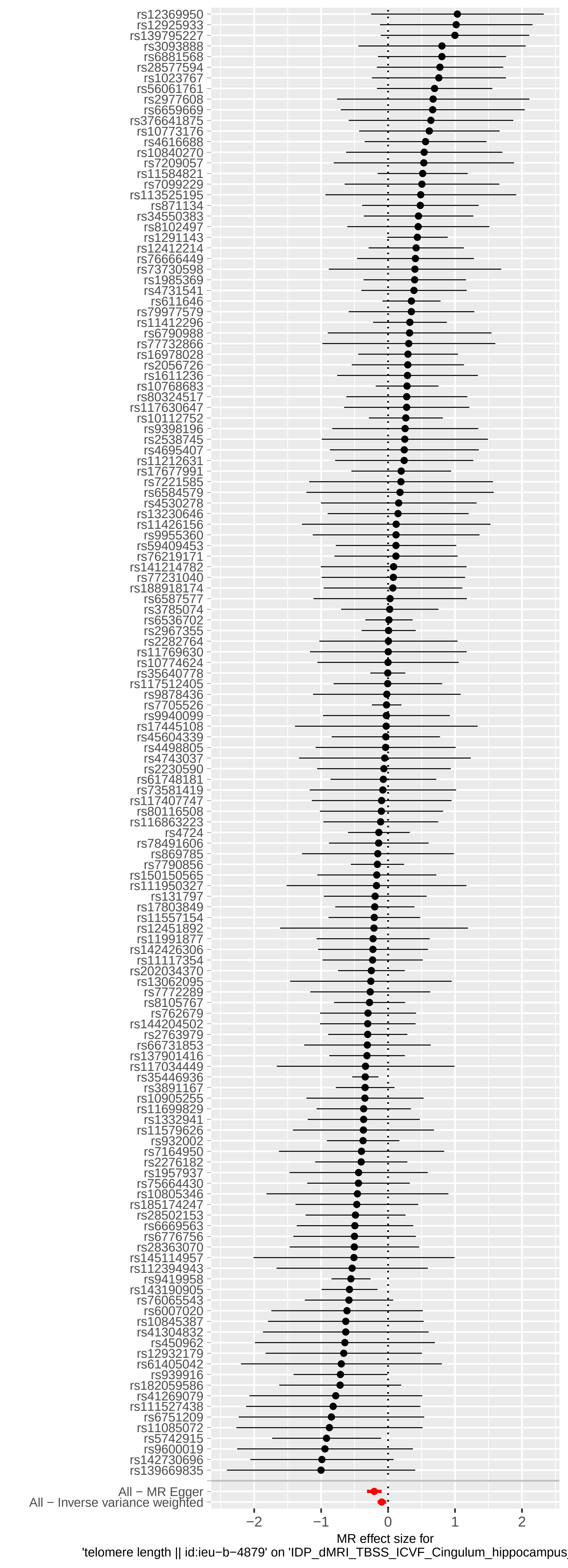
rs12369950
rs12925933
rs139795227
rs3093888
rs6881568
rs28577594
rs1023767
rs56061761
rs2977608
rs6659669
rs376641875
rs10773176
rs4616688
rs10840270
rs7209057
rs11584821
rs7099229
rs113525195
rs871134
rs34550383
rs8102497
rs1291143
rs12412214
rs76666449
rs73730598
rs1985369
rs4731541
rs611646
rs79977579
rs11412296
rs6790988
rs77732866
rs16978028
rs2056726
rs1611236
rs10768683
rs80324517
rs117630647
rs10112752
rs9398196
rs2538745
rs4695407
rs11212631
rs17677991
rs7221585
rs6584579
rs4530278
rs13230646
rs11426156
rs9955360
rs59409453
rs76219171
rs141214782
rs77231040
rs188918174
rs6587577
rs3785074
rs6536702
rs2967355
rs2282764
rs11769630
rs10774624
rs35640778
rs117512405
rs9878436
rs7705526
rs9940099
rs17445108
rs45604339
rs4498805
rs4743037
rs2230590
rs61748181
rs73581419
rs117407747
rs80116508
rs116863223
rs4724
rs78491606
rs869785
rs7790856
rs150150565
rs111950327
rs131797
rs17803849
rs11557154
rs12451892
rs11991877
rs142426306
rs11117354
rs202034370
rs13062095
rs7772289
rs8105767
rs762679
rs144204502
rs2763979
rs66731853
rs137901416
rs117034449
rs35446936
rs3891167
rs10905255
rs11699829
rs1332941
rs11579626
rs932002
rs7164950
rs2276182
rs1957937
rs75664430
rs10805346
rs185174247
rs28502153
rs6669563
rs6776756
rs28363070
rs145114957
rs112394943
rs9419958
rs143190905
rs76065543
rs6007020
rs10845387
rs41304832
rs450962
rs12932179
rs61405042
rs939916
rs182059586
rs41269079
rs111527438
rs6751209
rs11085072
rs5742915
rs9600019
rs142730696
rs139669835
All − MR Egger
All − Inverse variance weighted
−2
−1
0
1
2
MR effect size for
'telomere length || id:ieu−b−4879' on 'IDP_dMRI_TBSS_ICVF_Cingulum_hippocampus_
